# The Manchester Color Wheel: development of a novel way of identifying color choice and its validation in healthy, anxious and depressed individuals

**DOI:** 10.1186/1471-2288-10-12

**Published:** 2010-02-09

**Authors:** Helen R Carruthers, Julie Morris, Nicholas Tarrier, Peter J Whorwell

**Affiliations:** 1 Department of Medicine, University of Manchester, Manchester, UK; 2 Department of Medical Statistics, Wythenshawe Hospital, Manchester, UK; 3 Division of Clinical Psychology, School of Psychological Sciences, University of Manchester, Manchester, UK

## Abstract

**Background:**

For the purposes of our research programme we needed a simple, reliable and validated method for allowing choice of a color in response to a series of questions. On reviewing the literature no such instrument was available and this study aimed to rectify this situation. This was achieved by developing a simple method of presenting a series of colors to people validating it in healthy volunteers and in individuals where color choice might be distorted, namely anxiety and depression.

**Methods:**

A series of different presentations of four shades of eight colors and grey, as well as black and white were evaluated. 'Mood', 'favourite' and 'drawn to' colors were assessed in 105 healthy, 108 anxious and 110 depressed participants. The positive, neutral or negative attribution of these colors was recorded in a further 204 healthy volunteers.

**Results:**

The circular presentation of colors was most favoured (Color Wheel). Yellow was the most 'drawn to' color and blue the commonest 'favourite' color in all subjects. Yellow was most often associated with a normal mood and grey with an anxious or depressed mood. Different shades of the same color had completely different positive or negative connotations. Reproducibility was exceptionally high when color choice was recorded in positive, neutral or negative terms.

**Conclusions:**

The Color Wheel could be used to assess health status, mood or even treatment outcome in a variety of clinical situations. It may also have utility in circumstances where verbal communication may not be optimal, such as with children.

## Background

We have recently been studying the imagery of irritable bowel syndrome and shown that patients who have an image of their condition respond better to hypnotherapy than those who don't [1]. Furthermore, the response was even better if the image was in color. This has led us to speculate that how patients relate their illness or mood to color might be an area worthy of further investigation.

Colors are frequently used to describe emotions such as 'green with envy', 'red with rage' and being 'in the blues' when depressed. Although there is a large, often anecdotal, literature on color preferences [2-4] as well as the relationship of color to mood[5,6] and emotion[2,7,8] there has been relatively little systematic research on the subject [9-13]. Furthermore we could not find a single validated questionnaire specifically designed to rapidly identify color preferences in any previous investigations. When instruments have been developed such as the Color Pyramid test,[14] the Rorschach Inkblot test,[15] the Lüscher Color test,[16] the Lowenfeld Mosaic test[17] and the Stroop test[18] they have been designed more to interpret, for instance personality or cognitive processing, rather than allowing a subject to simply select a single color to represent their mood or disease.

It was therefore felt that it would be worth developing a color questionnaire which could present a reasonably wide range of colors in the form of a palette, similar to those used in paint charts, which would suit our purpose but may also have utility in a wide range of other areas of investigation and diagnosis. Validation was undertaken in normal individuals with respect to their 'drawn to', 'favourite' and 'mood' color choice with the purpose of identifying a 'normal range' of responses. In addition, anxious and depressed subjects were also studied as it was anticipated that their color choice might be distorted by their mood and this would aid the validation process by assessing discrimination between mood states. Furthermore, it was considered likely that different shades of the same color, for instance pale green and dark green, could have completely different connotations for the individual. Consequently, the positive and negative attributions of the colors in the questionnaire were also assessed as part of the validation process.

## Methods

### Preparation of material

#### 1. Choice of colors

**Figure 1:**
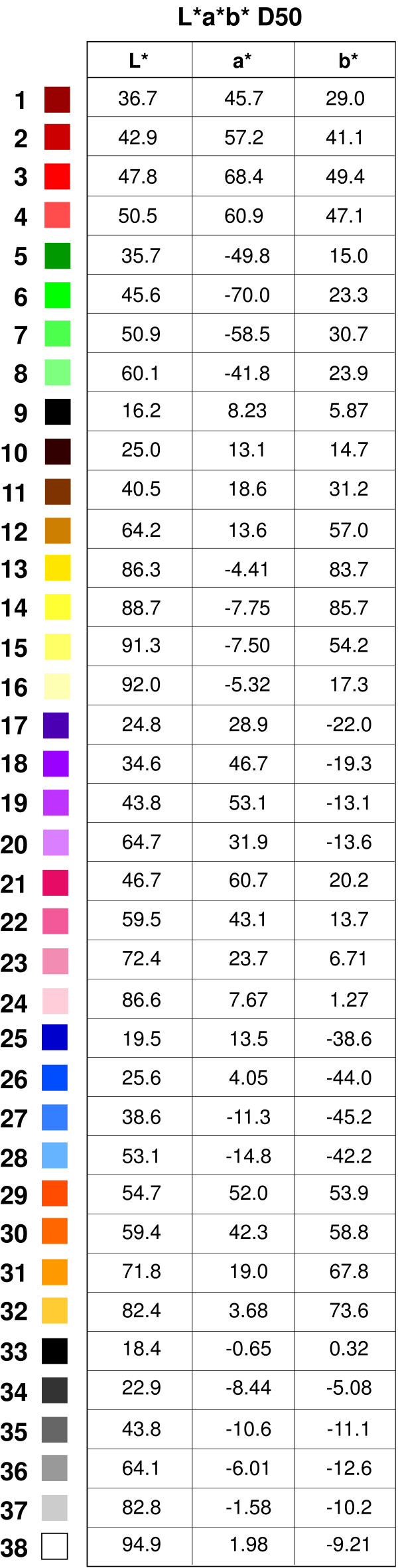
**Colors used in the Color Wheel**. Colors are shown with their corresponding L*a*b* D50 coordinates (CIE 1931; 2 degree Observer). White 38 also corresponds to the paper on which the Color Wheel was printed.

It was decided to include samples of six easily identifiable colors groups: red, orange, yellow, green, blue and purple. These colors correspond, at least in nomenclature, to the five principal color groups of the Munsell system, with the addition of orange as a separately identified color (instead of Munsell's Yellow-Red). It also seemed reasonable to use pink and brown as these might be related to bodily tissues and functions. Finally, we added black and white, two achromatic colors. It was felt that different shades of each color should be included as they may help the patient to discriminate the exact color of their choice. In addition dark versus light shades of a color might have different connotations for an individual. Thus four shades of each chromatic color and four additional shades of the achromatic color (grey shades) were included resulting in 38 colors in total. The colors with their L*a*b* D50 (CIE 1931; 2 degree Observer) coordinates are shown in Figure 1. The spectrums of the printed colors were measured with an i1-Pro spectrophotometer (X-Rite, Grand Rapids, Michigan, USA) and the spectral data processed according to the CIE Publication 15-2004 (Colorimetry, 3rd Edition) in order to compute the L*a*b* values shown in the figure.

#### 2. Presentation of colors

**Figure 2:**
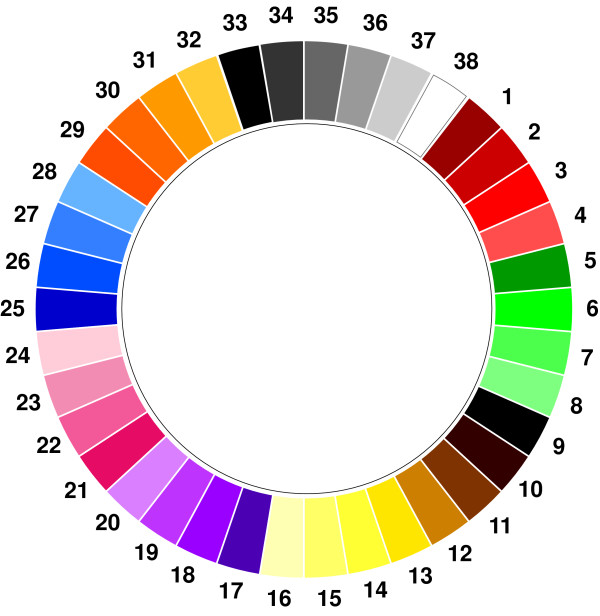
**The 'Color Wheel'**.

Five differing formats of presentation varying in shape and arrangement of colors (two linear, two tabular, one circular) were tested on three focus groups and the circular presentation, called the 'Color Wheel' (Figure 2), was the unanimous choice of the three groups. As can be seen from Figure 2, each color of the Color Wheel has been given a number to facilitate selection by those viewing the instrument as well as for the purposes of analysing the results. As some of the studies using the Color Wheel involved posting the questionnaire to participants, it was decided to use a printed version of the instrument rather than presenting it on a computer screen. To ensure homogeneity of color presentation all batches of the instrument were printed at the same time on bright-white paper. Like most bright-white papers, this paper contains optical brightening agents, which add some blue to the yellowish paper base, and which makes it look whiter. It was not possible to control for lighting conditions, but subjects were asked to complete the questionnaire under daylight conditions. Because the Color Wheel fills only a small portion of the printed page, and because the printed page covers a significant portion of the subject's field-of-view, the subject's vision adapts to the paper white, and all colors, chromatic and achromatic, are perceived in relation to this white. This relationship between the paper white and how the colors are perceived is important to maintain if the same colors are required in further studies. The measured paper white is actually the color used for white in the Color Wheel (White 38).

#### 3. Ethical approval

Ethical approval was sought and attained for all aspects of this study from the South Manchester Local Research Ethics Committee and all subjects gave written consent before participating.

### Study 1: 'Drawn to', 'favourite' and 'mood' colors with reproducibility in healthy volunteers

The main aim of this study was to devise a method of presenting colors to individuals in order to relate color choice to a particular question. For the purpose of validation, it was felt that 'favourite' color would be a reliable question as there is reasonably good existing evidence on 'favourite' colors. 'Mood' color would also provide an opportunity to study the instrument in a state where color choice is very likely to be consistently distorted, such as depression. 'Drawn to' color was suggested to us by several members of the focus groups and provided another parameter for checking consistency.

Following advertisement by global email to all employees at the University Hospital of South Manchester, 123 healthy volunteers (aged 22-70, mean age 42.2 years, 105 (85%) females, 18 (15%) males) were recruited and completed a color questionnaire using the Color Wheel. The following questions were asked: 1. Which color do you feel most drawn to? 2. Which is your favourite color? 3. With regard to your day-to-day mood over the last few months - do you associate it with a particular color? If so, which color? All participants were also asked to complete the Hospital Anxiety and Depression (HAD) Scale[19] so that those with significant anxiety and depression (score of 10 or greater for each domain) could be excluded. People suffering from any functional disorder or any other significant illness such as diabetes or heart disease were also excluded as were individuals with color blindness. 16 subjects had anxiety and 2 depression leaving 105 volunteers (aged 22-70 years, mean age 42.8 years, 90 (86%) females, 15 (14%) males) available for study. 59 of the volunteers were asked to repeat the study two weeks later to assess reproducibility. The percentage of respondents selecting an individual color in terms of it being their 'favourite' color, 'mood' color or the color they were 'drawn to' was calculated.

### Study 2: Positive and negative colors and their reproducibility in healthy volunteers

It was anticipated that there might be a wide variation in choice of colors when individuals were asked to select just one color in the first study. Furthermore, it seemed possible that different shades of the same colors may not necessarily have the same connotations for a particular person. Thus it may not be appropriate for the purposes of interpretation to group shades of the same color together and it could be that different shades of different colors may cluster better. It was therefore decided to study a further group of 255 healthy volunteers asking the following questions: 1. Which color or colors do you associate with a positive mood? 2. Which color or colors do you associate with a negative mood? Recruitment methods were identical to Study 1 as well as the inclusion and exclusion criteria of participants. Of the 255 individuals questioned 51 had anxiety and 5 depression (all 5 depressed subjects also had anxiety) on the HAD Scale[19] and were therefore excluded leaving 204 healthy volunteers (aged 18-72 years, mean age 38.5 years, 140 (69%) females, 64 (31%) males) for analysis. 59 participants were re-interviewed two weeks later to assess reproducibility. For each color the percentage of individuals rating it positive or negative was calculated.

### Study 3: Color choice in anxious and depressed individuals

Following advertisement in a local newspaper and by global email to all University of Manchester and University Hospital of South Manchester employees, 269 individuals claiming to be either anxious or depressed completed a color questionnaire with the aid of the Color Wheel via the post. They were also asked to complete the HAD Scale[19] to confirm the presence of anxiety or depression with a score above 9 for either anxiety or depression being indicative of significant morbidity. Of the respondents, 51 had either a HAD anxiety or depression score of 9 or less or were suffering from a functional disorder or another significant illness or color blindness. These individuals were excluded from the study leaving 108 anxious (aged 18-70 years, mean age 35.7 years, 86 (80%) females, 22 (20%) males) and 110 depressed individuals (aged 19-76 years, mean age 40.6 years, 74 (67%) females, 36 (33%) males) for the analysis.

All patients were asked: 1. Which color do you feel most drawn to? 2. Which color is your favourite color? 3. With regard to your day-to-day mood over the last few months - do you associate it with a particular color? If so, which color? Is there a reason why you have chosen this color? Comparisons were made with data obtained for healthy controls in Study 1. In addition to analysing the data regarding the individual color choices, the responses in terms of positive, neutral and negative colors were also documented. This allowed the behaviour of the different permutations to be compared between healthy, anxious and depressed individuals.

### Statistical analysis

The percentage of respondents selecting an individual color in terms of it being their 'favourite' color, 'mood' color or the color they were 'drawn to' was calculated.

The statistical package SPSS 11.5 was used for analysing the data. Parametric tests were used for the Normally distributed data. The distribution of data was assessed by calculating skewness and kurtosis statistics. The Pearson Chi-squared test was used to assess relationships between categorical variables and the Fisher's Exact test where the volunteer numbers were small. ANOVA was used to compare the mean values of anxiety and depression with the individual color groupings of each permutation with regard to mood and multiple comparisons were carried out using the Scheffé Post-hoc test[20] on the same data.

Non-Normally distributed data were analysed using Spearman's correlation, for example, when assessing the relationship between the mean anxiety scores and the various color groupings.

## Results

### Study 1

With regard to 'drawn to' color 104 (99%) of the 105 healthy volunteers answered this question with 14 (14%) participants choosing 'Yellow 14' as their most popular 'drawn to' color. 103 (98%) subjects answered the question regarding 'favourite' color with 16 (16%) choosing 'Blue 28'. Not one volunteer chose white or grey. With respect to 'mood' color only 41 (39%) of the healthy volunteers associated it with a color with 'Yellow14' being the most popular 'mood' color choice.

#### Reproducibility

In order to assess reproducibility 59 healthy volunteers repeated the color questionnaire after an interval of two weeks. 20 (34%) individuals gave exactly the same 'drawn to' color response, 27 (46%) chose the same 'favourite' color and 38 (64%) gave the same 'mood' color.

### Study 2

**Figure 3:**
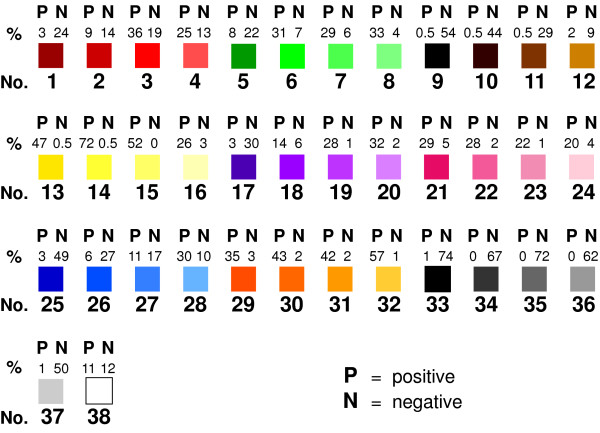
**Positive and negative ratings for each color by healthy volunteers**. The percentage of healthy volunteers who rated each color on the 'Color Wheel' as either positive (P) or negative (N) is shown. For example, 72% of participants rated Yellow 14 as positive whereas 0.5% rated it negative in contrast only 1% of volunteers rated Black 33 as positive whereas 74% rated it as negative.

**Figure 4:**
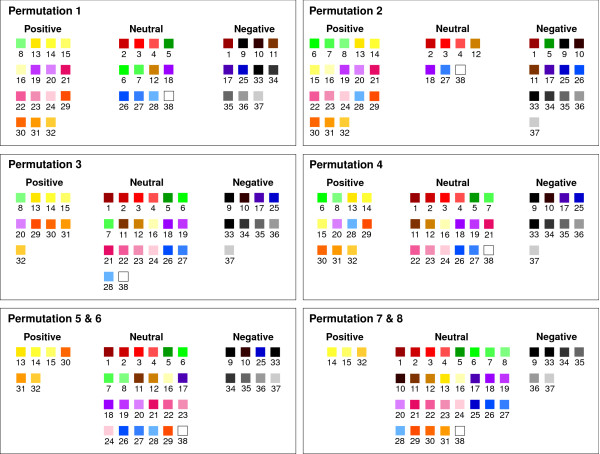
**Distribution of colors throughout the eight permutations**. The construction of the eight permutations is fully described in Table 1. Permutations 1 and 2 are the most inclusive of positive and negative colors whereas Permutations 7 and 8 are the most restrictive. If a shade was considered neither positive nor negative in a particular permutation it was classified as neutral.

The percentage of individuals rating a particular color as positive or negative is shown in Figure 3. For instance, color number 1 was considered positive by 3% and negative by 24% whereas color number 30 was considered positive in 43% and negative by 2%. Thus there was considerable variation in the positive and negative attributions of the different colors. It was therefore necessary to establish what combination of percentages, both positive and negative, best gave an indication of whether a color should definitely be regarded as positive or negative with the remainder being classified as neutral. Consequently a series of eight permutations were constructed (Table 1) using varying percentages for what was judged a positive or negative color. For example, Permutation 1 defined a positive color as one in which 20% or more of individuals regarded it as positive and 5% or less regarded it as negative. Similarly, a color was defined as negative if 20% or more of individuals rated it as negative and 5% or less rated it as positive. Any colors not meeting these criteria were classified as neutral. Subsequent permutations became increasingly more restrictive. The distribution of colors in each of the different permutations is detailed in Figure 4. A further striking finding was that colors from the same color group (i.e. blues or reds) had completely different connotations for individuals, in terms of their positivity or negativity, depending on brightness and saturation (i.e. dark purple versus pale purple). Consequently, similar shades from different color groups, for instance dark greens and dark reds, were more likely to have the same connotation than different shades from the same color group.

**Table 1:** Construction of the eight permutations.

Permutation 1	Positive color = ≥20 (pos) and ≤ 5 (neg)
	Negative color = ≥20 (neg) and ≤ 5 (pos)

**Permutation 2**	Positive color = ≥20 (pos) and ≤ 10 (neg)

	Negative color = ≥20 (neg) and ≤ 10 (pos)

**Permutation 3**	Positive color = ≥30 (pos) and ≤ 5 (neg)

	Negative color = ≥30 (neg) and ≤ 5 (pos)

**Permutation 4**	Positive color = ≥30 (pos) and ≤ 10 (neg)

	Negative color = ≥30 (neg) and ≤ 10 (pos)

**Permutation 5**	Positive color = ≥40 (pos) and ≤ 5 (neg)

	Negative color = ≥40 (neg) and ≤ 5 (pos)

**Permutation 6**	Positive color = ≥40 (pos) and ≤ 10 (neg)

	Negative color = ≥40 (neg) and ≤ 10 (pos)

**Permutation 7**	Positive color = ≥50 (pos) and ≤ 5 (neg)

	Negative color = ≥50 (neg) and ≤ 5 (pos)

**Permutation 8**	Positive color = ≥50 (pos) and ≤ 10 (neg)

	Negative color = ≥50 (neg) and ≤ 10 (pos)

In Permutation 1 a color was defined as positive if 20% or more of individuals regarded it as positive and 5% or less regarded it as negative. Similarly, a color was regarded as negative if 20% or more of individuals regarded it as negative and 5% or less of individuals regarded it as positive. Subsequent permutations used varying degrees of positivity and negativity.

#### Reproducibility

In order to assess reproducibility 23 individuals repeated the color questionnaire two weeks later. Out of a total of 874 possible responses regarding choosing positive mood colors, 767 (88%) were exactly the same. Similarly, 798 (91%) negative mood colors were identical when asked a second time.

### Study 3

**Figure 5:**
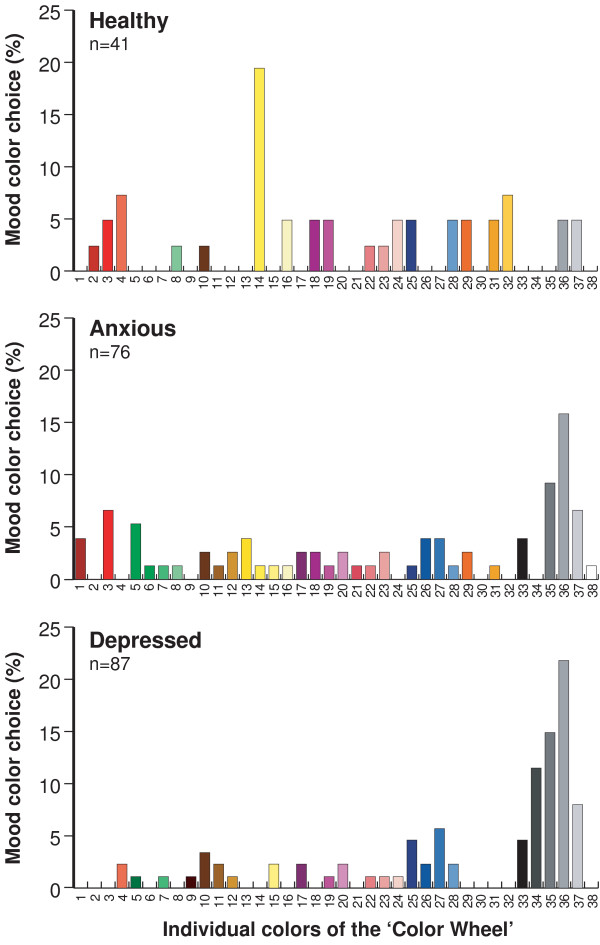
**Distribution of 'mood' color choice in healthy, anxious and depressed individuals**. 41 (39%) healthy subjects, 76 (70%) anxious and 87 (79%) depressed volunteers related color to their mood. Compared with healthy volunteers, the choice of 'Yellow 14' is significantly reduced in both depressed (p < 0.001) and anxious (p = 0.001) individuals (Fisher's Exact test). In contrast, compared with healthy volunteers, the choice of 'Grey 36' is significantly increased in depressed (p = 0.020) individuals but not in those with anxiety (p = 0.134) (Fisher's Exact test). Volunteers not choosing a color to describe their mood have been omitted from the figure.

108 (100%) anxious and 108 (98%) depressed individuals answered the 'drawn to' question with 'Yellow 14' being the most popular in both groups (anxious = 13 (12%), depressed = 11 (10%)). 108 (100%) anxious and 109 (99%) depressed subjects responded to the question on 'favourite' color with 11 (10%) anxious choosing 'Blue 28' as the most popular color and 18 (17%) depressed choosing 'Blue 27' closely followed by 15 (17%) individuals choosing 'Blue 28'. 76 (70%) anxious and 87 (79%) depressed volunteers related a color to their mood with 'Grey 35' and 'Grey 36' monopolising the top two places of these two groups. 12 (16%) anxious individuals and 19 (22%) depressed chose 'Grey 36' and 7 (9%) anxious and 13 (15%) depressed chose 'Grey 35' (Figure 5).

#### Differences between the healthy, anxious and depressed volunteers with regard to 'mood' color

It was thought that analysing the results of the healthy, anxious and depressed individuals would help to determine which of the permutations described in Study 2 would be the most reliable and robust for use in future studies. However, since there were no differences between the three groups of participants with regard to 'drawn to' and 'favourite' colors, it was unnecessary to assess this with respect to positive and negative colors. In contrast, there were striking differences with regard to color choice and mood between the three groups of volunteers. 87 (79%) depressed, 76 (70%) anxious and only 41 (39%) healthy subjects answered the question on mood and color with the difference between healthy and depressed subjects being highly significant (Chi-square (1) = 35.756; p < 0.001). The percentage of participants choosing an individual color in relation to their mood is shown in Figure 5. As can be seen, 'Grey 36' was the most common color chosen by the anxious and depressed groups in contrast to 'Yellow 14' in the healthy volunteers. It is noteworthy that healthy individuals tended to choose saturated colors, the anxious were largely random and the depressed group largely chose desaturated colors.

#### Comparison of color choice between the eight permutations of 'mood' colors in healthy, anxious and depressed volunteers

Table 2 compares the positive, neutral and negative color choice in those healthy, anxious and depressed volunteers choosing a color to describe their mood. As can be seen, there were highly significant differences between color choice and the three volunteer groups in all eight permutations (p < 0.001). Depressed individuals showed a striking preference for negative colors choosing relatively few positive colors when compared to healthy controls. Anxious individuals gave results intermediate to those observed in depression with negative colors being chosen more frequently as well as positive colors being chosen less frequently. All specific comparisons between healthy versus anxious and healthy versus depressed revealed a significance of p < 0.005.

**Table 2:** Comparison of'mood' colour choice in the healthy, anxious and depressed volunteers for each permutation.

Permutation number	Color choice		Volunteer group		p value
		**Healthy** **(n = 41)** ***No. (%)*** **respondents**	**Anxious** **(n = 76)** ***No. (%)*** **respondents**	**Depressed** **(n = 87)** ***No. (%)*** **respondents**	

	positive	24 (59%)	17 (22%)	8 (9%)	Chi-square (4)

1	neutral	10 (24%)	23 (30%)	14 (16%)	= 50.158;

	negative	7 (17%)	36 (47%)	65 (75%)	p < 0.001

					

	positive	26 (63%)	20 (26%)	11 (13%)	Chi-square (4)

2	neutral	8 (20%)	13 (17%)	8 (9%)	= 46.633;

	negative	7 (17%)	43 (57%)	68 (78%)	p < 0.001

					

	positive	16 (39%)	11 (15%)	4 (5%)	Chi-square (4)

3	neutral	18 (44%)	33 (43%)	20 (23%)	= 46.442;

	negative	7 (17%)	32 (42%)	63 (72%)	p < 0.001

					

	positive	18 (44%)	13 (17%)	6 (7%)	Chi-square (4)

4	neutral	16 (39%)	31 (41%)	18 (21%)	= 45.660;

	negative	7 (17%)	32 (42%)	63 (72%)	p < 0.001

					

	positive	13 (32%)	6 (8%)	2 (2%)	Chi-square (4)

5 & 6	neutral	21 (51%)	40 (53%)	24 (28%)	= 49.491;

	negative	7 (17%)	30 (40%)	61 (70%)	p < 0.001

					

	positive	11 (27%)	2 (3%)	2 (2%)	Chi-square (4)

7 & 8	neutral	26 (63%)	47 (62%)	31 (36%)	= 52.899;

	negative	4 (10%)	27 (37%)	54 (62%)	p < 0.001

Comparison of healthy versus anxious and healthy versus depressed was also significant (p < 0.005) for all permutations.

#### Relationship between eight permutations of positive, neutral and negative 'mood' colors and mean anxiety and depression scores in healthy, anxious and depressed volunteers

**Figure 6:**
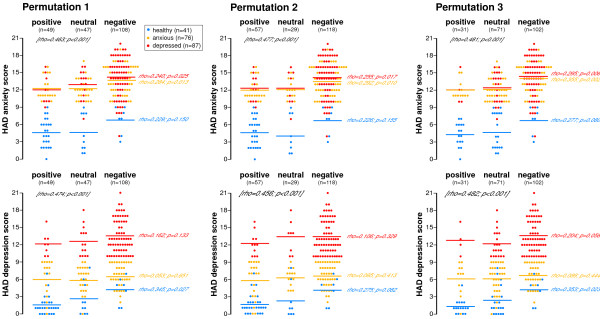
**Relationship between mean anxiety and depression scores and 'mood' color (positive, neutral or negative) in healthy, anxious and depressed volunteers in Permutations 1-3**. Healthy subjects are shown in blue, anxious in orange and depressed in red. The appropriately colored dots indicate each individual's HAD anxiety (top row of figures) or depression (bottom row of figures) score and the lines of a similar color show each groups mean score. The rho values written in black, represent the overall magnitude of the relationships of the three groups namely, healthy, anxious and depressed whereas the rhos written in red, orange and blue represent the individual groups. Optimal permutations were identified by those which best discriminated between the various subgroups and which displayed a greater strength of relationship between the degree of anxiety/depression and positive/negative colors for example, Permutation 7 & 8 were most marked in HAD depression in healthy volunteers (p = 0.008) and Permutation 4 was most marked for HAD anxiety in the anxious group (p = 0.001). Data was analysed using Spearman's correlation.

**Figure 7:**
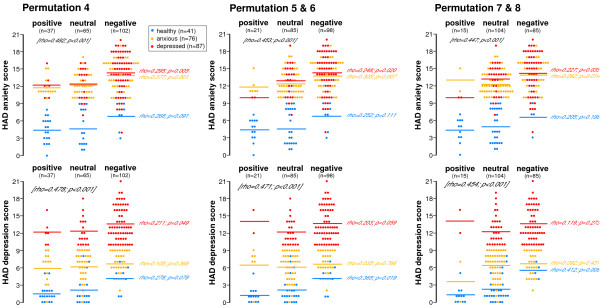
**Relationship between mean anxiety and depression scores and 'mood' color (positive, neutral or negative) in healthy, anxious and depressed volunteers in Permutations 4-8**. Healthy subjects are shown in blue, anxious in orange and depressed in red. The appropriately colored dots indicate each individual's HAD anxiety (top row of figures) or depression (bottom row of figures) score and the lines of a similar color show each groups mean score. The rho values written in black, represent the overall magnitude of the relationships of the three groups namely, healthy, anxious and depressed whereas the rhos written in red, orange and blue represent the individual groups. Optimal permutations were identified by those which best discriminated between the various subgroups and which displayed a greater strength of relationship between the degree of anxiety/depression and positive/negative colors for example, Permutation 7 & 8 were most marked in HAD depression in healthy volunteers (p = 0.008) and Permutation 4 was most marked for HAD anxiety in the anxious group (p = 0.001). Data was analysed using Spearman's correlation.

To further attempt to understand the relationship between mood and color choice (positive, neutral or negative) the results for healthy volunteers as well as individuals with anxiety or depression were all plotted together for each permutation (Figures 6 and 7). This form of presentation gave an instant impression of the different color choices by the different groups of individuals.

#### Healthy volunteer group

On first impression, it appears that the mean anxiety scores for healthy individuals in Figures 6 and 7 were higher for those choosing a negative color than for those choosing a positive or neutral color. However this did not reach statistical significance. In contrast, depression scores in healthy volunteers did relate to positive, neutral and negative color choice and reached significance for all permutations except 2 and 4 which showed a trend in the same direction. Those with higher scores for depression, even though they were still within the normal range, were more likely to choose a negative color and this effect was most marked in Permutations 7 and 8 (rho = 0.412; p = 0.008).

#### Anxious volunteer group

In anxious volunteers, the mean anxiety scores did relate to positive, neutral and negative color choice and reached significance for all permutations. Those with higher scores for anxiety were more likely to choose a negative color and this effect was most marked in Permutation 4 (rho = 0.377; p = 0.001). In contrast, the mean depression score for anxious individuals was similar irrespective of whether they chose a positive, neutral or negative color as determined by any of the permutations.

#### Depressed volunteer group

The mean depression scores in the depressed volunteer group related to color choice and reached significance in Permutation 4 (rho = 0.211; p = 0.049) with Permutations 3, 5 and 6 also showing trends in the same direction. Likewise, the mean anxiety scores were significantly related to positive, neutral and negative color choice irrespective of permutation. The higher the anxiety score the more likely they were to choose a negative color and this effect was greatest in Permutation 4 (rho = 0.295; p = 0.005).

#### Relationship between eight permutations of positive, neutral and negative 'mood' colors as well as no color choice at all and mean anxiety and depression scores in healthy, anxious and depressed volunteers

The opportunity was also taken to compare anxiety and depression scores in individuals choosing positive, neutral and negative colors and compare these with individuals choosing no color at all. This was undertaken in order to ascertain whether individuals choosing no color differed in any way from the others.

#### Healthy volunteer group

The scores in individuals who did not choose a color appeared to approximate to those observed in subjects choosing positive or neutral colors. However, the anxiety and depression scores for those not choosing a color appeared to be lower than in those choosing negative colors and in the case of the depression scores the difference was always significant irrespective of permutation. This difference was most marked in Permutations 7 and 8 (p = 0.005; negative color: mean = 5.5; CI = 3.4, 7.6 and no color at all: mean 1.8; CI = 1.4, 2.3). (The results were expressed as mean 95% confidence intervals).

#### Anxious volunteer group

In all permutations the anxiety and depression scores for those not choosing a color were lower than in those choosing negative colors with the difference for anxiety scores always reaching significance and being most marked in Permutation 4 (F(3,104) = 4.692; p = 0.004). The mean depression scores in individuals who did not choose a color approximated to those observed in subjects choosing positive or neutral colors.

#### Depressed volunteer group

The anxiety and depression scores for those not choosing a color were lower than those choosing neutral and negative colors and reached significance in the case of the anxiety scores, irrespective of permutation. This difference between individuals choosing a negative color or no color at all was most marked in Permutations 3 and 4 (F(3,106) = 5.644; p = 0.001 and F(3,106) = 5.633; p = 0.001 respectively). The mean depression scores were also significantly different with respect to color choice with this difference being most marked in Permutations 5 and 6 (F(3,106) = 5.201; p = 0.002).

## Discussion

Colors are all about us and the sheer variety of shades used in, for instance interior decorations, are an indication of the wide range of preferences that exist. This study describes a novel way of documenting the color preferences of individuals which is extremely easy to use.

In addition to studying healthy volunteers it was felt necessary to also include a group of individuals in whom color choice might be distorted. It was hypothesised that people with depression would be most likely to show a definite effect between color and mood as even in every day life dark colors are often regarded as depressing. Individuals with anxiety were also studied to test the discriminant power of using color to define mood. In other words, if anxious individuals selected different colors it would suggest that color choice is identifying a specific state of mind rather than just reflecting a more general change in mood.

There was remarkably little difference in choice of 'drawn to' or 'favourite' color between individuals irrespective of whether they were healthy, anxious or depressed. 'Yellow 14' was the most popular 'drawn to' color and with respect to 'favourite' color, 'Blue 28' was the most popular color choice of healthy and anxious individuals with 'Blue 27' being the first choice of the depressed subjects closely followed by 'Blue 28'. These results are in accord with the findings of previous investigators who similarly have found that blue was the favourite color choice of adults[2-4,8,21-23]. It is noteworthy that only two individuals chose a grey shade or white as their favourite color.

The results for mood color were in stark contrast to those obtained for 'drawn to' and 'favourite' color. Only 39% of healthy volunteers associated a color with their mood compared to 70% of anxious and 79% of depressed which suggests that individuals with affective disorders are more likely to equate their mood with a color. 'Grey 35', 'Grey 36' and 'Grey 37' dominated the top four places of the anxious and depressed groups, a theme broken only by 7% of anxious individuals who chose 'Red 3' to describe their mood likening it to anger, stress and frustration. Comments about the choice of grey included relating it to a dark state of mind, a colorless and monotonous life, gloom, misery or a disinterest in life rather than the color grey being chosen because of its colloquial associations with a low mood. These descriptions are in keeping with the observations of others suggesting that depressed patients viewed life as "monochromatic"[24] or having "lost its color"[25]. It has even been suggested that depressed people might exhibit color impairment and that it might be a state or trait marker of mood disorders[26]. The results for the anxious and depressed individuals differed considerably to those in healthy volunteers who associated 'Yellow 14' and the yellow colors in general as the color most representative of their mood. Another noteworthy observation was that anxious and depressed individuals were significantly less likely to choose 'Yellow 14' to describe their mood and significantly less likely to choose colors from either the red, orange or yellow color groupings. In the literature yellow has been reported as being associated with happiness, cheerfulness and a positive emotional state[5,6,27,28]. It has been suggested that differences in color saturation and brightness are associated with different emotional feelings[29] and it is possible that this could provide an alternative explanation for the different mood color responses that have been observed in this study. For instance, healthy individuals may relate their mood to saturated colors whereas depressed subjects choose desaturated colors. Color choice was not related to gender in the group as a whole and this is in accord with the older literature which suggests that although it may be different in children this is lost with adulthood. However, there were more women in all of the study groups which may have reflected the method of recruitment by advertisement and global e-mail, although anxiety and depression are more common in females, which is a more likely explanation for the gender imbalance. Consequently it was necessary to ensure the gender distribution of the healthy group matched that of the other two groups so that the gender distribution was approximately equal across the whole study. A more recent study has suggested there may be differences in color choice between males and females[30] but this needs to be confirmed and does not strictly affect the validation of the instrument, which could actually be used to investigate this question in the future, especially in larger samples. The use of antidepressants in those with anxiety or depression did not appear to affect color choice. The lens of the eye tends to become more yellow with age and this could theoretically affect color choice. However there is evidence that this is compensated for, possibly by neural mechanisms,[31] and consequently this effect was not looked for especially as the number of elderly subjects was relatively small.

It was felt that reproducibility would be an important part of the validation exercise to ensure that choice was not just a random process. Consequently a sample of subjects from both Study 1 and 2 were asked to repeat the questionnaire two weeks later. This interval was selected because it was felt that color choice should remain fairly constant over this period of time, but it was long enough for them to forget their original choice. Some interesting observations emerged when reproducibility was examined. With respect to 'drawn to', 'favourite' and 'mood' color the reproducibility, 34%, 46% and 64% respectively, might initially appear to be rather poor. However, it was noteworthy that even if an individual did not choose exactly the same color on the second occasion they often chose one closely related on the color spectrum, e.g. 'Red 3' on the first and 'Pink 21' on the second. If the data are assessed using this criterion then reproducibility increases to 59%, 78% and 76% respectively. Another critical finding in this study was that the shade of a color was a very important factor. Consequently color groupings, such as reds, greens and yellows may not be a good way of assessing color preferences. This is because it became clear from this study that a pale shade of a color may have a completely different connotation to a much darker shade. For instance, dark blue may have similar mood or emotional connotations to dark brown rather than to a pale shade of blue. These problems can be overcome by adopting the alternative methodology of classifying a color as either positive or negative. When this was undertaken reproducibility reached extremely high values of 88% and 91% respectively for positive or negative shades suggesting that this approach may be much more robust than relying on individual color choice.

When colors were assessed according to their positive and negative associations it was observed that there was a tendency for some colors, such as yellow or black, to be far more commonly rated as either positive or negative than others. Thus, it was possible to create a variety of permutations which at one extreme were restricted to just the most highly rated positive or negative colors such as Permutation 8. Alternatively, a much more balanced permutation containing equal numbers of positive, neutral and negative colors such as Permutation 4 resulted from attributing a positive or negative connotation to a far greater number of colors. It may be that for some studies colors with a very high positive or negative rating may be more appropriate whereas in other investigations a broader range of somewhat lower rated colors may be a better option.

The effect of anxiety and depression on the different permutations of positive, neutral and negative colors was assessed. As can be seen from Figures 6 and 7, individuals with anxiety and depression did show different patterns of color choice compared to each other as well as healthy subjects. These figures also show that negative colors are more likely to be chosen by depressed subjects and that in most cases the higher the depression score the more likely they are to choose such a color. Another notable finding was that even in healthy volunteers with depression scores within the normal range those with higher scores more frequently chose a negative color.

It is important that if the Color Wheel were to be used to help identify depression, that the majority of patients would need to choose a mood color for it to be effective in the clinical situation. However, our results indicate that a decline in mood does seem to provoke individuals to choose a color and even in depressed individuals who did not choose a color, the anxiety and depression scores were significantly lower than in those who chose a negative color irrespective of permutation. This suggests that as anxiety and depression gets worse the individual is more likely to choose a color to describe their mood. Therefore, the more anxious or depressed a person is, the more the Color Wheel approach is likely to identify the problem. Similar trends were also observed in healthy and anxious individuals although the differences did not always reach significance. The other remarkable observation from looking at Figures 6 and 7 is that it highlights the way there is a stepwise increase in anxiety and especially depression scores as individuals choose more neutral and then negative color shades. This seems to occur irrespective of whether individuals are healthy, anxious or depressed although it is most marked for healthy subjects and least apparent in the anxious and depressed group. However, it should be noted that the depressed individuals already had relatively high scores and therefore a further stepwise increase in score would be less likely to occur as they were already scoring highly.

Another aim of this study was to try and determine which permutation would be the best for use in future investigations. Regarding the relationship between mean anxiety and depression scores and positive, neutral and negative color choice, Permutation 4 was the best at detecting depression in truly depressed individuals. Likewise, Permutation 4 was also best at detecting higher anxiety scores in both anxious and depressed individuals. Permutations 7 and 8 performed best at detecting higher, although in the normal range, depression scores in healthy volunteers but did not perform well with regard to anxiety scores in this group of individuals. It therefore appears that Permutations 4, 7 and 8 are the most effective permutations although the latter two have a very restricted color choice and only work well in healthy individuals. It is probably better to choose the permutation that works best for those with a distorted color choice and thus Permutation 4 with its wider, more balanced distribution of positive, neutral and negative colors and which works well both in anxious and depressed patients would appear to be the best choice.

With regard to future studies, the Color Wheel approach could be used in children to detect affective disorders, as communication using color might be easier for them to understand than lengthy questionnaires that contain words they may not readily comprehend. The instrument would have to be revalidated in children as their response to color may differ to that of adults. The Color Wheel could also be used to help people with communication problems or those in whom English is not their first language. However, since the majority of the population examined in this study were white and of British origin, the results may not be readily transferable to groups from different cultural backgrounds. For example, in some Eastern cultures there is a strong preference for the color white[32] which is in contrast to what was found in this study where white was seldom chosen. Thus the Color Wheel may need to be validated in different ethnic groups and this might even possibly reveal divergences in color preferences that hitherto may not have been recognised. Lastly, it has to be born in mind that many psychologically orientated questionnaires are designed to detect either a normal or negative situation. In contrast, the Color Wheel is an instrument that not only can do that but also has the ability to identify a positive state of affairs.

## Conclusion

The Color Wheel provides an easy to use method of assessing color choice in relation to a variety of clinical situations and has the added advantage that it dispenses with the need for verbal communication.

## Abbreviations

CIE: Commission Internationale de l'Eclairage; HAD: Hospital Anxiety and Depression Scale.

## Competing interests

The authors declare that they have no competing interests.

## Authors' contributions

HRC participated in the design of the study, collected and analysed all the data as well as drafting the manuscript. JM advised on the statistical analysis of the data and checked the accuracy of the final results. NT participated in the design of the study, acted as PhD supervisor to HRC and reviewed and critiqued the manuscript. PJW conceived the project and participated in its design as well as finalising the manuscript. All authors have read and approved the final manuscript.

## Pre-publication history

The pre-publication history for this paper can be accessed here:

http://www.biomedcentral.com/1471-2288/10/12/prepub
